# Dissolution Testing of Hardly Soluble Materials by Surface Sensitive Techniques: Clotrimazole from an Insoluble Matrix

**DOI:** 10.1007/s11095-014-1368-5

**Published:** 2014-04-22

**Authors:** Heike M. A. Ehmann, Sascha Winter, Thomas Griesser, Roman Keimel, Simone Schrank, Andreas Zimmer, Oliver Werzer

**Affiliations:** 1 Institute of Pharmaceutical Sciences, Department of Pharmaceutical Technology, University of Graz, Universititätsplatz 1, 8010 Graz, Austria; 2 Institute of Physical and Theoretical Chemistry, University of Graz, Heinrichstraße 28, 8010 Graz, Austria; 3 Chemistry of Polymeric Materials, Montanuniversität Leoben, Otto-Glöckelstrasse 2, 8700 Leoben, Austria; 4 Institute for Process and Particle Engineering, University of Technology Graz, Inffeldgasse 13, 8010 Graz, Austria

**Keywords:** clotrimazole, composite, contact angle, differential scanning calorimetry, dissolution, drug release, polystyrene, quartz crystal microbalance with dissipation, thin film, X-ray photoelectron spectroscopy, X-ray reflectivity

## Abstract

**Purpose:**

The low aqueous solubility of many drugs impedes detailed investigation as the detection limit of standard testing routines is limited. This is further complicated within application relevant thin films typical used in patches or stripes for buccal or topical routes.

**Methods:**

In this work a model system is developed based on spin – casting technique allowing defined clotrimazole and clotrimazole – polystyrene composite films preparation at a solid surface. Various highly sensitive techniques including quarz crystal microbalance (QCM), X-ray reflevtivity (XRR) and X-ray photon spectroscopy (XPS) are used to investigate the drug release over time into an aqueous media.

**Results:**

The results reveal a steady drug release for both samples over the course of the experiments but with the release from the composite being significantly slower. In addition the dissolution rate of the clotrimazole sample initially increases up to 30 min after which a decrease is noted. XRR shows that this is a result of surface roughening together with film thickness reduction. The results for the composite show that the release in the composite film is a result of drug diffusion within the matrix and collapsing PS film thickness whereby XPS shows that the amount of clotrimazole at the surface after 800 min immersion is still high.

**Conclusion:**

It can be stated that the applied techniques allow following low mass drug release in detail which may also be applied to other systems like pellets or surface loaded nano-carriers providing information for processing and application relevant parameters.

## INTRODUCTION

The demand for smart drug delivery systems has grown exponentially in the last decades as well as proper characterization techniques to investigate the stability and drug release behavior. Drug release from polymeric composite thin films has become a hot topic in the field of transdermal and buccal pharmaceutical applications with strongly enhanced bio-availablitiy (1–6). The ability for multilayer film sandwiches, each slice loaded with different drug materials, promise highly tunable combinatorial medications (7). This approach allows to build-up drug delivery systems based on thin films, where the release of specific drugs in a certain time-scale can be adjusted by adopting the solubility of either a biocompatible polymer/drug composite layer, or the drug itself which may be dispersed within an insoluble polymer matrix. The high bioavailablity results in low amounts of drug molecules being required for a therapeutic action. An important parameter for the bioactivity as well as *in vitro* and *in vivo* dissolution of drugs is the stabilization of the amorphous active pharmaceutical ingredient (API) *via* a proper matrix, to inhibit the nucleation of the drug (8–10). However, small loads of drug loading results in standard test methods like UV/V is spectroscopy being at their limit; in both qualitative and quantitative means. In this sense, a variety of experimental methods is required to allow gaining sufficient information on the dissolution properties of such systems.

Within this work the usage of X-ray reflectivity (XRR), quartz crystal microbalance with dissipation (QCM-D) and X-ray photoelectron spectroscopy (XPS) is applied to investigate the dissolution of clotrimazole thin films in contrast to films, where clotrimazole is dispersed within a polystyrene (PS) matrix. Clotrimazole is widely used in fungal treatments and shows promising properties within the treatment of other diseases (11). Clotrimazole is a hardly water soluble drug molecule (0.49 mg/L) (11) which makes it a perfect candidate for this study. XRR is a scattering based technique allowing the layer thickness, density and surface roughness to be accurately determined (12–14). QCM-D experiment is capable of determining mass changes with a sensitivity of ngcm^−2^ as it was proven in several publications (15,16). Furthermore changes in the dissipative responds of the crystal on film changes (mass change, swelling, …) allow interactions to be elucidated (17,18). XPS is a highly surface sensitive technique enabling chemical composition within the surface near region, about 10 nm from the air – solid interface, to be determined. On that notes, these highly sensitive methods are combined to investigate the elusive drug release properties of clotrimazole containing thin films.

## EXPERIMENTAL SECTION

### Materials

Clotrimazole of pharmaceutical grade was purchased from Gatt-Koller GmbH and polystyrene (PS; M_w_ ~ 100 kDa) was purchased from Aldrich and used without further purification. As substrates thermally grown silicon oxide surfaces (purchased from SilChem) were used and cleaned in acetone, isopropanol and a NaOH solution and finally treated with ozone revealing highly hydrophilic surfaces. Toluene solutions containing 2.0 wt% PS, 2.0 wt% clotrimazole and a composite solution containing 1.0 wt% of each (PS and clotrimazole, respectively) were prepared and spin coated onto substrates at a spin speed of 25 rps for 30 s. After spin coating the films were dried for 1 h at 27°C under vacuum. All dissolution experiments were performed in Milli-Q water at 21°C.

### Differential Scanning Calorimetry (DSC)

DSC was performed in a DSC 204 F1 Phoenix (Netzsch, Selb, Germany). Defined masses ranging between 5 and 10 mg were placed into aluminum pans *via* drop casting the solution and after solvent evaporation this were closed with a pierced lid. Samples were investigated in a temperature range between 30 and 180°C at a heating rate of 10°C/min under nitrogen atmosphere.

### Dynamic Light Scattering (DLS)

Dynamic light scattering (DLS) measurements were performed using a Malvern Zetasizer NanoZS (Malvern, USA). The experiments were carried out at 25°C and the solutions were equilibrated for 2 min, before each measurement and the backscattering (at 173°) was recorded.

### Quartz Crystal Microbalances with Dissipation (QCM-D)

Measurements were performed on a Q-Sense E4 (Q-Sense, Gothenborg, Sweden) at 21°C. Au coated quartz crystals (GSX301 with nominal 5 MHz resonant frequency, Q-Sense, Gothenburg, Sweden). Dissolution was tested under constant flow rate of 0.1 mL/min. All experiments were repeated two times using two chambers simultaneously. For the mass desorption the third overtone (Δ*f*_3_) was applied to the Sauerbrey mass (19) formula:

1 $$ \varDelta m=-\frac{C\cdotp \varDelta \mathrm{f}}{n} $$

whereby *Δm* is the Sauerbrey mass, *C* is the Sauerbrey constant (17.7 ng Hz^−1^ cm^−2^), Δ*f* the observed frequency shift and *n* is the overtone number. Changes in the film viscoelasticity result in oscillations of the crystal which are not fully coupled with the film itself. However, all measurements reveal nearly constant dissipation values during the course of the experiments and are omitted within this manuscript.

### Specular X-ray Reflectivity (XRR)

Measurements were performed with a Panalytical Empyrian Reflectometer equipped with a copper sealed tube (*λ* = 0.154 nm), a Goebel mirror and slits. A 3d – Xpert detector collected the scattered intensity from the sample. The data are represented in scattering vector notation *q*_*z*_ = 4*pi sin(θ)/λ with *θ* being the incident angle. Data evaluation was performed with the software package WinGIXA (Philips) providing information on the layer thickness, electron density and the roughness of the films by applying Parrats formalism (20) and the disturbance term of Nevot– Croce (21).

### X-ray Photoelectron Spectroscopy (XPS)

XPS spectra were recorded with a monochromatic K_α_ spectrometer equipped with an Al source (1486.6 eV) operating with a base pressure in the range of 10^−8^ to 10^−10^ mbar. Wide scans were acquired with a pass energy of 100 eV and a step size of 1.0 eV. All spectra have been normalized to the Au 4f7/2 peak. Charge compensation was performed with an argon flood gun. The average chemical composition was calculated from wide scan spectra in two different locations on each surface. The peaks were fitted using a Gaussian/Lorenzian mixed function employing Shirley background correction. All analyses were performed at room temperature.

### Contact Angle (CA)

CA measurements were performed using the OCA15+ setup from Dataphysics with a 6-fold zoom lens optics with integrated fine focus (± 6 mm) and camera. For the CA measurement Milli-Q water was used in sessile drop configuration (volume of 3 μL/drop). The drop size was steadily increased (advancing) for the avoidance of gravitational effects and to elucidate the impact of the roughness (22).

## RESULTS

### Differential Scanning Calorimetry

**Fig. 1:**
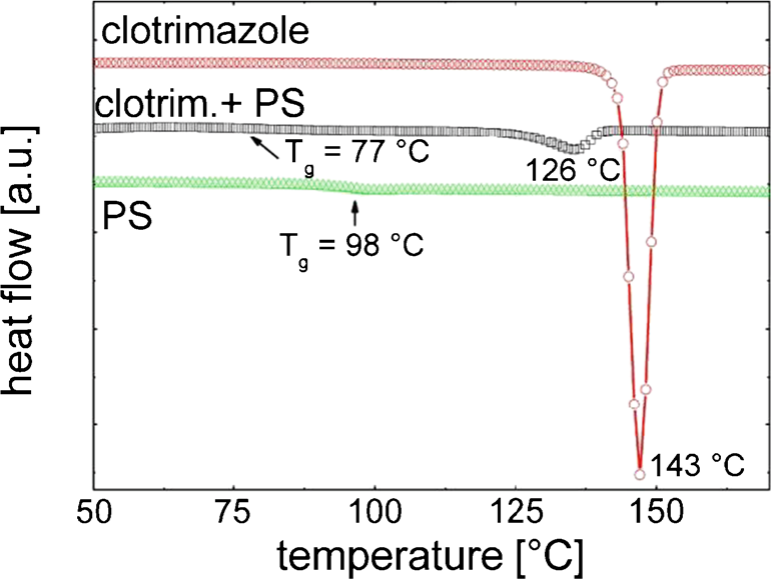
DSC measurements of clotrimazole, polystyrene (PS) and the PS-Clot composite on increasing heat. For the sake of clarity curves are shifted by means of their y-axis.

Polystyrene, clotrimazole and a composite with a 1:1 ratio of clotrimazole and PS were drop casted and dried. The drop casted films contrarily to the spin coated films crystallize very fast (in the first 30 min), while the spin coated films are amorphous over several weeks (23). Anyway, for the identification of PS – clotrimazole interactions this kind of sample provides some insight even thought the other films are amorphous. The first heating run at a heating rate of 10°C/min is shown in Fig. 1. The drug free PS sample shows a glass transition (*T*_*g*_) at 98°C. The polymer free clotrimazole sample has a melting peak at 143°C. The polystyrene –clotrimazole composite reveals that *T*_*g*_ and the melting point of the one-component systems have changed. *T*_*g*_ shifted towards lower temperatures to 77°C. The melting point present in the clotrimazole sample has shifted by 17°C down to 126°C. This shows that both, *i.e.* clotrimazole and PS, are affected by the intermixing.

The crystallization of clotrimazole within the pure and the composite material was not observed on cooling. As the crystallization of clotrimazole is slow, the relatively fast cooling rate of 5°C/min is not sufficient for the recrystallization of the clotrimazole within these experiments.

### Dynamic Light Scattering

**Fig. 2:**
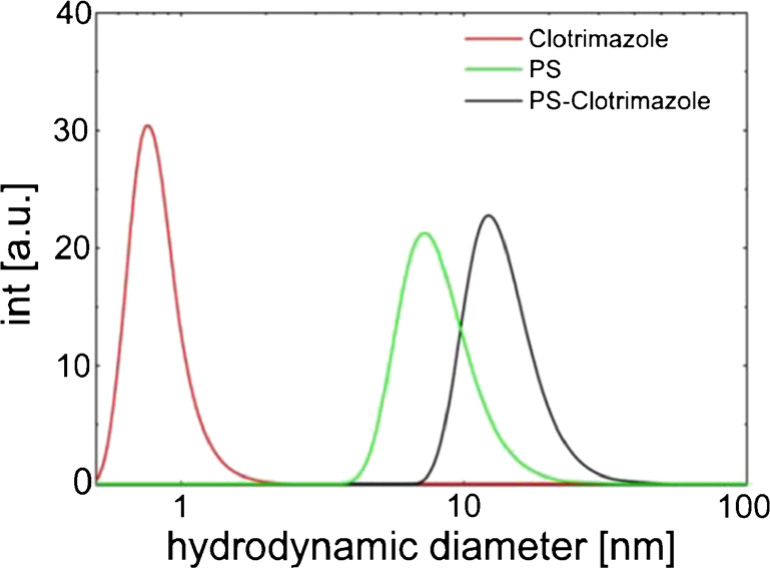
Dynamic light scattering measurements of PS (*green*), clotrimazole (*red*) and PS-clotrimazole (*black*) in toluene.

The dynamic light scattering (DLS) measurements in Fig. 2 reveal the number weighted particle size of PS (green line), clotrimazole (red line) and PS-clotrimazole (black line) in toluene. It can be seen that clotrimazole has a very small hydrodynamic diameter (d ~ 1 nm; close to the detection limit), while the PS polymer coil has a mean diameter of 7.2 nm. The mixture of both components results in a total number weighted particle dimension of 12.1 nm. This value is remarkably larger compared to the sum of the single measurements, which indicates that the excess of clotrimazole by means of the polystyrene polymer (M_w_ ~ 100 kDa) leads to a swelling of the polymer coil within the solution or the formation of clusters is promoted by clotrimazole.

### Quartz Crystal Microbalance with Dissipation

**Fig. 3:**
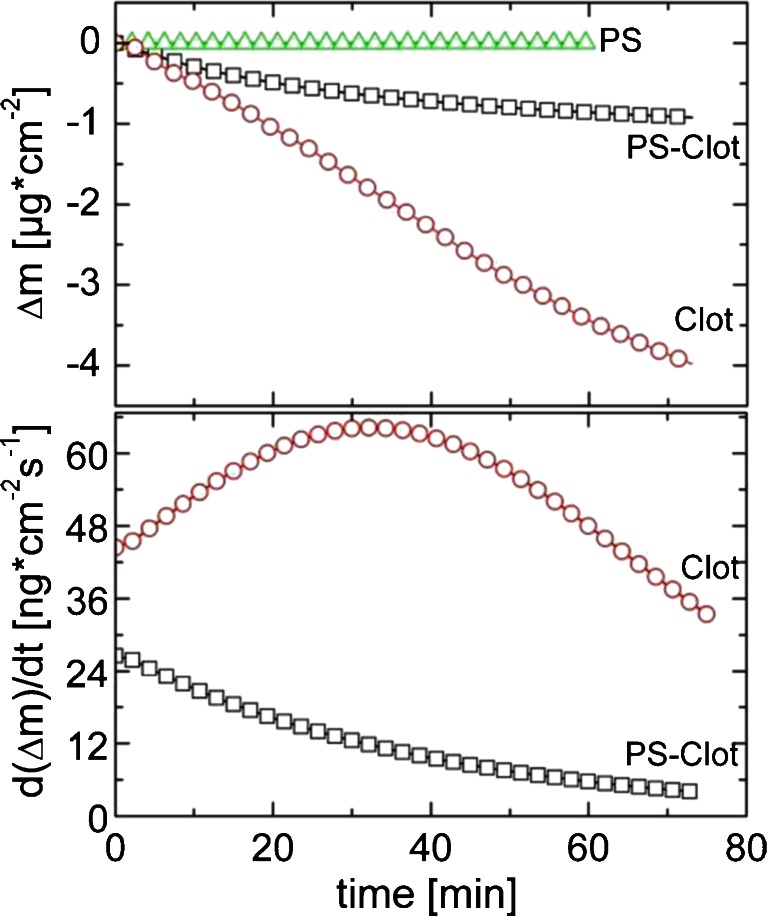
Sauerbrey mass loss (*Δm*) as function of the water rinsing time of various films (**a**). Corresponding time derivatives over the rinsing time of same signals (**b**). Both graphs are plotted on the same x-axis for comparability.

Within a QCM-D experiment the change in resonance frequency with mass load change is determined allowing the desorption from the surface being followed. In Fig. 3a the relative Sauerbrey mass losses of three different thin film samples are shown as function of MilliQ-water rinsing times. The pure PS thin film shows a constant frequency over time which means that this film is not affected by the continuous rinsing. However, the pure clotrimazole film reveals a different behavior and shows a typical trajectory (nearly exponential decay) for a controlled desorption from a solid surface with a steady change of mass load with rinsing time. After a time of 73 min. the corresponding mass loss is 3.9 μg cm^−2^. Similarly, the immersion of the PS-Clot composite film reveals a steady mass loss, but the trajectory of the dissolution profile (now a Boltzmann function like decay) and the maximum mass loss is significantly different; after 73 min only 0.9 μg cm^−2^ is lost.

In Fig. 3b the time derivative of the mass loss is plotted. Such a plot shows changes in desorption rates over time, *i.e.* the mass desorption in a constant time frame. The plot for the pure clotrimazole film (squares) shows an initial desorption rate of 44 ng cm^−2^ s^−1^ and increases nearly linearly as time progresses. At 32 min the desorptionrate reaches a maximum; a rate of 64 ng cm^−2^ s^−1^ is observed. After this maximum, the desorption rate starts to decrease and a nearly linearly behavior is observed for measurement times larger than 48 min. As the experiment was performed under continuous flow conditions, the flow changes in the desorption profile must be a result from changes of the sample and are not a result from solution saturation; *i.e.* the exchange of solvent prevents solvent saturation. The time derivative of the composite PS-Clot (circles) is distinct to the behavior of the pure clotrimazole film. Initially a rate of 27 ng cm^−2^ s^−1^ is present. Other than for the pure clotrimazole film the rate change is decreasing with time rather than increasing. At long rinsing times the rate change flattens, *i.e.* the curve approaches asymptotically a value around 3.7 ng cm^−2^ s^−1^.

Within all experiments the dissipative investigation revealed nearly constant values in the limit of detection. From this follows that swelling of the film if present, is of minor importance for the samples under investigation.

### X-Ray Reflectivity

For the investigation of the morphological changes on immersion time, *ex-situ* XRR measurements are performed. The measurements of the PS film deposited from a 2 wt% solution onto a silica surface reveal a homogenous film with a layer thickness of 55 nm and surface roughness of about 2 nm (data not shown). Immersion of such a film for 800 min and subsequent drying under a nitrogen stream did not change any film properties in accordance with the QCM-D experiments showing that the PS film on the silica surface remains unaffected by the water treatment.

**Fig. 4:**
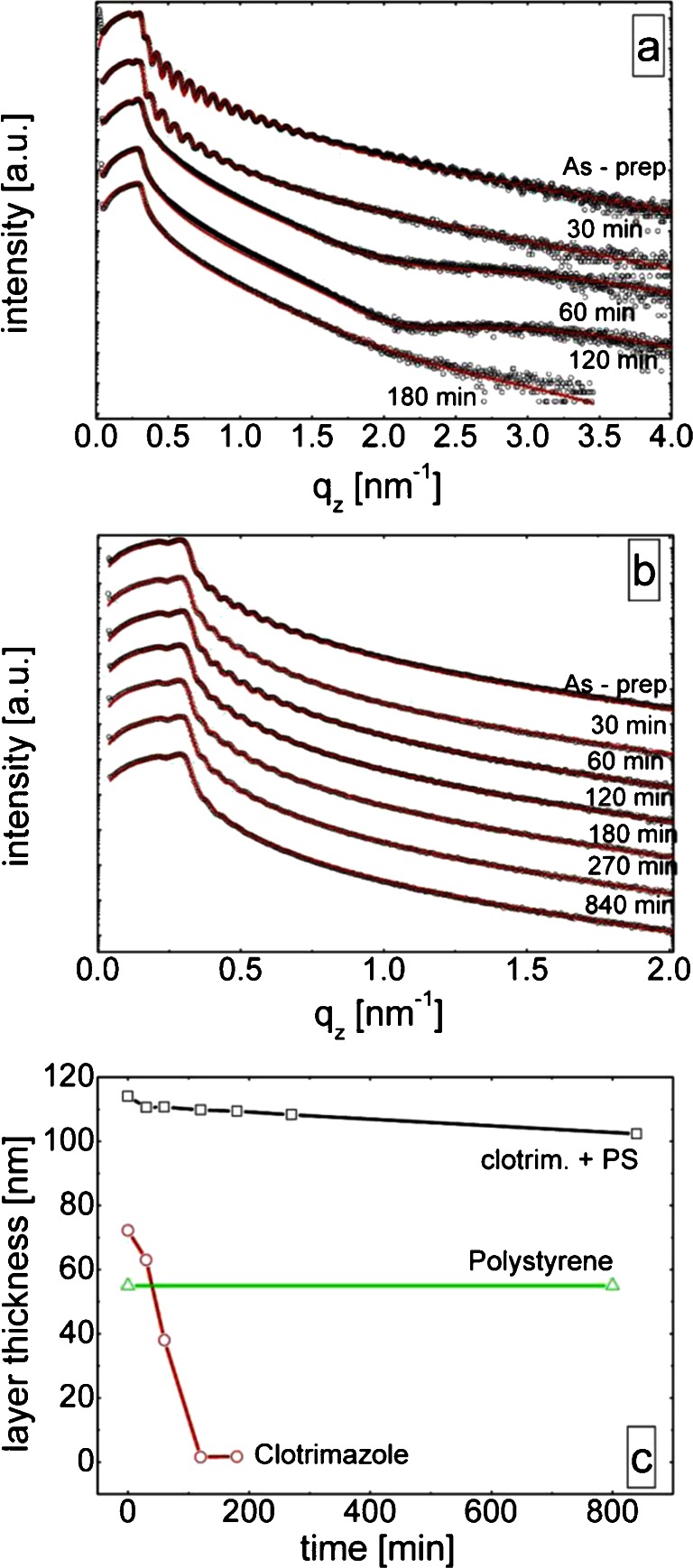
Scattered intensities as a function of the scattering vector for a pure clotrimazole film (**a**) and for a clotrimazole – PS composites film (**b**) after several immersion times. Curves are shifted for clarity. The extracted layer thicknesses as a function of time (**c**).

The results of the reflectivity investigations of the clotrimazole thin film as function of the immersion time are shown in Fig. 4a. At low scattering vectors up to *q*_*z*_ = 0.33 nm^−1^ nearly constant intensity is observed. At slightly higher scattering vectors a strong decrease in the intensity starts. The point at which the decrease is significant is the critical scattering vector of the silica surface at *q*_*z*_ = 0.33 nm^−1^; X-ray beams illuminating the surface at shallower scattering vectors are not able to penetrate the substrate as total external reflection occurs. For scattering vectors much larger than the critical scattering vector, the intensity falls according to the Fresnel reflectivity at a solid substrate following *I ~ q*_*z*_^−4^. Besides the changes in intensity the curve reveal minima and maxima (Kiessig fringes) (24) which allow extracting information from the films by using model fits with Parratts formalism (20). The fits reveal an average clotrimazole film thickness of 72 nm after the deposition. The root mean square surface roughness (σ_rms_) obtained from this fit is 1.6 nm. After an immersion time of 30 min of the pure clotrimazole film in water and drying under a nitrogen stream the XRR curve markedly changed. The width of the oscillation (or distance from minima to minima) is increased compared to the as-prepared film. This is typical for a decreased layer thickness being present; the fits reveal that the thickness has reduced to 63 nm (compare Fig. 4c). The scattering vector up to which oscillations are present within the XRR curve is reduced to *q*_*z*_ = 1 nm^−1^ compared to 1.8 nm^−1^ from the as-prepared film and is a consequence of an increase in the surface roughness to 2.7 nm.

After an immersion time of 60 min the XRR does not show any fast oscillation and only one minimum at *q*_*z*_ = 2 nm^−1^ remains. In addition the curve shows the presence of a critical scattering vector for the organic thin film prior *q*_*z*_ = 0.33 nm^−1^. From this the layer thickness of the layer can be approximated to be 38 nm. The film is rough but as no fast oscillations are present, reliable values cannot be given but it is expected that the roughness is larger than 2.7 nm, *i.e.* after 30 min immersion. After 120 min immersion, the XRR curves remain very similar but the edge of the critical wave vector of the organic layer is vanished. This shows that most of the organic material is dissolved. The fit reveals a thin layer of clotrimazole is still present; a layer thickness of 1.7 nm and a surface roughness of 0.4 nm is determined. This suggests that this layer consists of a monolayer which, on the account of molecule – surface interaction, remains during the desorption process of the bulk at the interface. Another hour of immersion is required to partially dissolve this layer; at 180 min an incomplete monolayer with a coverage of about 25% is present.

Similar to the QCM-D experiments, the XRR measurements of the clotrimazole – PS composite film show a distinct behavior compared to the pure clotrimazole film. The measurement of the as – prepared film reveal a homogeneous film with a layer thickness of 114 nm (see Fig. 4b and c) and a surface roughness of about 4 nm. While the concentration of the composite of PS and clotrimazole is half compared to the “pure” samples, the layer thickness is markedly higher. This is a result of interactions between clotrimazole and PS resulting in a higher viscosity within the toluene solution and thus thicker films during the spin coating form.

The water immersion leads to a decrease in film thickness in the first 30 min by 3.5 nm to 110.5 nm, while the surface roughness remains the same. The film thickness decreases further steadily with the immersion time. Compared to the results obtained from the pure PS film, where the layer thickness was constant even after an immersion time of around 800 min, it can be concluded that the film thickness decrease in the composite is solely due to clotrimazole release. The rate of layer thickness reduction with increasing immersion time decreases; the layer thickness after 180 and 270 min is 109.4 nm and 108.3 nm, respectively. At a total immersion time of 840 min the layer thickness is still 102.4 nm which means that the overall layer thickness reduction is just 10% over this long immersion time.

### X-ray Photoelectron Spectroscopy

XPS was used to identify the amount of clotrimazole in the vicinity of the surface. Within an as - prepared clotrimazole film in the absence of PS an amount of 4.7 at.−% of chlorine (related to the total amount of carbon) was detected using the Cl2p peak (201.57 eV). As the clotrimazole is dispersed within the PS matrix, the peak intensity decreases, which reveals that the chlorine content decreased to around 1 at.−%. Thus, the XPS analysis reveals a significant amount of clotrimazole being present at the solid - air interface of the PS - clot composite. After 800 min immersion the clotrimazole concentration is reduced to 0.7 at.−% which shows that the API is still present at the surface after this long immersion time.

### Contact Angle

Contact angle measurement with MilliQ-water, performed on the polystyrene thin film, reveal a contact angle of 98.1 ± 1.4° meaning that the surface is of highly hydrophobic nature. The pure clotrimazole thin film has a contact angle of 77.7 ± 1°, which suggests that the affinity of the clotrimazole to make contact with the water is slightly larger compared to the pure PS film. The as – prepared composite reveal a contact angle of 99.1 ± 2.6° which is the same, within errors, compared to the pure PS film. This suggests that the PS dominates the surface properties with the composite after preparation. Surprisingly, as the film is immersed for 90 h within water, the contact angle reduces to 88.0 ± 1.73° suggesting that the clotrimazole molecules arrange within the surface more effectively resulting in the contact angle lying between those of pure clotrimazole and pure PS.

## DISCUSSION

The preparation of the clotrimazole thin film and the composite results in amorphous thin films as a spin coating process was used. However for the preparation of a sample for the thermal analysis a drop casting process was used which induces crystal growth of clotrimazole (25). A thermal analysis of a sample using a film that is scratched from the surface, typically used, is not suitable as mechanical stress induced crystal growth. Anyway, the DSC investigation give some insight in interaction that take place as clotrimazole is in contact with the PS matrix.

The temperature investigation with DSC reveal a melting point of pure clotrimazole to be around 143°C while it is lowered as clotrimazole is hosted in the PS matrix and shifted to 126°C. Similarly, the glass transition temperature (*T*_*g*_) of PS shifts towards lower values in the composite films; *i.e.*, from 98 to 77°C. Lowering of the melting event frequently indicates the formation of an eutectic system (26,27) and could suggest interactions of clotrimazole with the PS on the molecular level (28). However, it must be noted that clotrimazole and PS did not form a solid solution as the heating scan of composite films still showed an endothermic peak correlating to the melting of clotrimazole and a glass transition of PS which corresponds to softening of the PS. There is an ongoing debate about the *T*_*g*_ and the influence of the polymer film thicknesses (29,30). It is stated that in ultrathin polymer films the *T*_*g*_ can either increase (30,31) or decrease (31,32) with decreasing film thickness, even if the latter one has drawn much more attention due to the often bigger size of the effects. However, the layer model is the most cited approach to describe the thickness dependency of *T*_*g*_, which indicates that the thin films consists of a highly mobile surface layer on the top of a less mobile (bulk-like) layer (29–32). We assume, on the one hand, that some fractions may be completely dissolved into one another which form a partially eutectic system of clotrimazole and polystyrene due to the dominating hydrophobic interactions. This is further supported by DLS results which show a swelling of the polystyrene polymer coils in solution on the addition of clotrimzole. But on the other hand fractions in the sample may still consist of only one component (domains), which indicates that they are not completely mixed at the molecular level (amorphous solid dispersion).

Dissolution testing of pure PS films reveals that water does not affect the film properties, *i.e.* the polystyrene is insoluble in water and is not affected by immersion or continuous flow conditions. From this follows that material desorbing from the surface within the course of the experiments must be a consequence of clotrimazole molecules dissolving into the liquid media. Small amount of PS – clotrimazole clusters may be disconnected from the surface but all experimental data suggest that this is of minor importance.

**Fig. 5:**
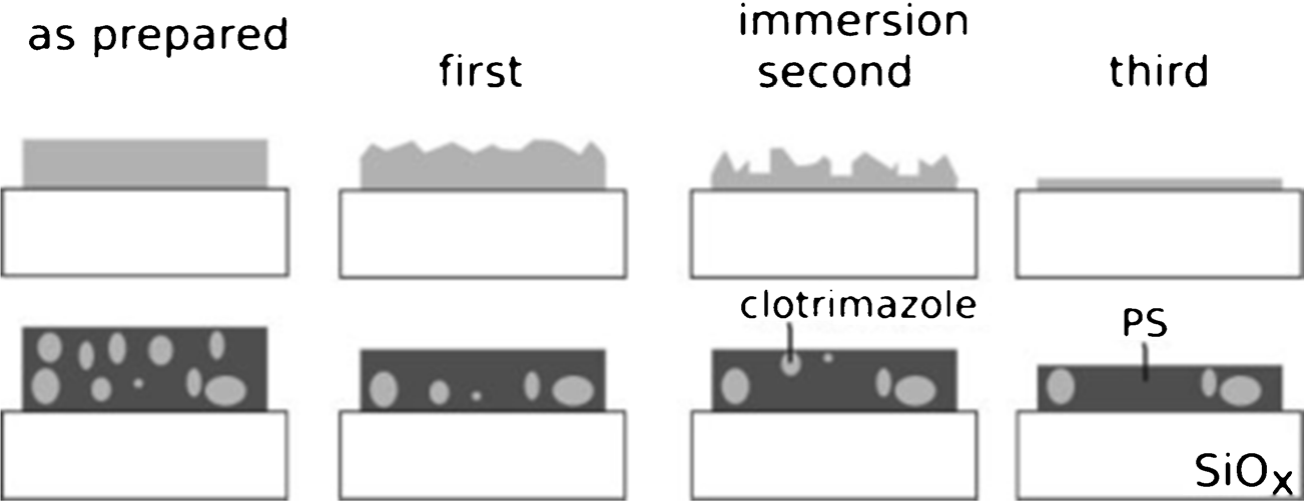
Sketch of the dissolution process of pure clotrimazole (*top*) and the composite (*bottom*).

The combination of all techniques allows to follow the dissolution process of the pure drug substance and within the composite in detail. The pure clotrimazole film shows a steady decreasing mass load within the QCM-D experiment which causes a reduction in the layer thickness as observed in the XRR measurement. The time resolved measurements reveal that the dissolution is not linear with time. Furthermore an increase in the desorption rate is observed as time progresses (compare Figs. 3b and 4c). For the sake of simplicity a scheme is shown in Fig. 5 providing information on the morphological changes during the dissolution process. As the overall layer thickness decreases and the surface roughness increases (compare Fig. 5 top second from the left), *i.e.* the depth of the valleys in the film increases, the suggestion arises that the desorption process is isotropic. Surface molecules exposed to water desorb with the desorption probability along hills and valleys being equally likely (33). A rough and jagged surface has a larger surface area compared to a smooth flat surface. From this follows according to Noyes-Whitney that dissolution is enhanced; QCM-D measurements suggest that a maximum surface area is present after about 38 min.

After the maximum surface area is reached the valleys reach the silica – clotrimazole interface. Besides the strong roughening, the XRR measurement reveals that at this stage a fully closed monolayer remains between the bulk clotrimazole and the silica surface. The surface area providing desorption sites for the clotrimazole starts to decrease and a reduction in the dissolution rate results. The rough bulk layer dissolved continuously until a fully closed monolayer remains at the silica surface. Long immersion times are required to desorb molecules from this monolayer. Even at an immersion time of 180 min 25% of the surface is covered with monolayer fragments thus interactions of the clotrimazole monolayer and the substrate surface are strong; molecules favor the contact with the glass surface rather than the contact with the polar aqueous environment due to their hydrophobic nature.

The dissolution behavior is markedly changed on the incorporation of the clotrimazole in to the PS matrix. The contact angle measurements reveal that the PS is the dominant species at the composite – water interface. This is in excellent agreement with the XPS results which shows that the amount of Cl-atoms close to the surface is reduced from 4.7 at.−% in the pure clotrimazole sample down to about 1.0 at.−% on the addition of PS. However, dissolution of clotrimazole over the course of the experiments takes place. Initially a fast dissolution is observed; clotrimazole close to the composite – liquid interface rapidly dissolves into the bulk liquid (Fig. 5 bottom left). Dissolving clotrimazole molecules results in vacancies in the composite film. These most likely collapse which results in the overall layer thickness of the composite being reduced as determined by XRR. After the initial fast dissolution, the rate of mass loss reduces. After interfacial clotrimazole is dissolved additional clotrimazole molecules need to diffuse within the matrix up to the composite – liquid interface to be able dissolving into the bulk liquid. This diffusion process is slow. The diffusion distance that the clotrimazole molecules have to travel to reach the surface increases with progressing immersion time, thus the dissolution rate markedly decreases with time. The XRR measurements reveal that even after an immersion time of 800 min the layer thickness of the film changes which suggests that clotrimazole is still present within the sample. In fact, XPS measurement reveal that the initial amount of Cl-atoms at the surface from about 1 at.−% dropped to 0.7 at.−% after this long immersion time. Surprisingly, the contact angle measurement of the composite after the immersion reveals a reduction by 10° which suggest that the clotrimazole is equally likely at the interface with respect of the PS chains. From this follows that the overall amount of clotrimazole is reduced within the surface area but that clotrimazole molecules most likely arrange in favor of a contact with the water. The nitrogen atom in clotrimazole is able to provide hydrogen bonding sites while the Cl-atoms are highly hydrophobic which suggest that a preferred orientation form during the immersion process.

## CONCLUSION

In this work it is demonstrated that modern analytical approaches are suitable to characterize the qualitative as well as quantitative release of hardly water soluble drugs in the submicro-range. DSC, QCM-D, XRR, XPS and CA measurements, respectively, reveal complementary information on the dissolution behavior of clotrimazole within thin films of pure and composite films. The change in the dissolution of the bulk clotrimazole film on the silica surface results from film roughening; isotropic dissolution properties cause an increase in the surface area thus provide more desorption sites for the dissolution process. The addition of PS into the sample prevents a significant surface roughening. Furthermore diffusion of the clotrimazole molecules within the matrix to the surface is required to achieve dissolution. Both effects results in the drug dosage which can be applied over time being reduced. However, even after 800 min of immersion time clotrimazole is still able to escape from the composite bulk which makes this approach a perfect candidate for an application form with a controlled release.

## ABBREVIATIONS

CA: Contact angle
Clot: Clotrimazole
DLS: Dynamic light scattering
DSC: Differential scanning calorimetry
PS: Polystyrene
PS-Clot: Polystyrene-clotrimazole
QCM-D: Quartz crystal micro balance with dissipation
XPS: X-ray photoelectron spectroscopy
XRR: X-ray reflectivity

## References

[1] Ariga K, Hill JP, Ji QM (2008). Biomaterials and biofunctionality in layered macromolecular assemblies. Macromol Biosci.

[2] Stuart MAC, Huck WTS, Genzer J, Muller M, Ober C, Stamm M (2010). Emerging applications of stimuli-responsive polymer materials. Nat Mater.

[3] Ozaydin-Ince G, Gleason KK, Demirel MC (2011). A stimuli-responsive coaxial nanofilm for burst release. Soft Matter.

[4] Roblegg E, Jager E, Hodzic A, Koscher G, Mohr S, Zimmer A (2011). Development of sustained-release lipophilic calcium stearate pellets via hot melt extrusion. Eur J Pharm Biopharm.

[5] Amorosi C, Ball V, Bour J, Bertani P, Toniazzo V, Ruch D (2012). One step preparation of plasma based polymer films for drug release. Mat Sci Eng C-Mater.

[6] Roblegg E, Frohlich E, Meindl C, Teubl B, Zaversky M, Zimmer A (2012). Evaluation of a physiological in vitro system to study the transport of nanoparticles through the buccal mucosa. Nanotoxicology.

[7] Chen D, Chen J, Wu M, Tian H, Chen X, Sun J (2013). Robust and flexible free-standing films for unidirectional drug delivery. Langmuir.

[8] Lamm MS, Simpson A, McNevin M, Frankenfeld C, Nay R, Variankaval N (2012). Probing the effect of drug loading and humidity on the mechanical properties of solid dispersions with nanoindentation: antiplasticization of a polymer by a drug molecule. Mol Pharm.

[9] Newman AW, Byrn SR (2003). Solid-state analysis of the active pharmaceutical ingredient in drug products. Drug Discov Today.

[10] Blagden N, de Matas M, Gavan PT, York P (2007). Crystal engineering of active pharmaceutical ingredients to improve solubility and dissolution rates. Adv Drug Deliv Rev.

[11] Prabagar B, Yoo BK, Woo JS, Kim JA, Rhee JD, Piao MG (2007). Enhanced bioavailability of poorly water-soluble clotrimazole by inclusion with beta-cyclodextrin. Arch Pharm Res.

[12] Werzer O, Resel R (2013). Model-independent X-ray reflectivity fitting for structure analysis of poly(3-hexylthiophene) films. Macromolecules.

[13] Werzer O, Stadlober B, Haase A, Flesch HG, Resel R (2009). Evaluation of organic sub-monolayers by X-ray based measurements under gracing incident conditions. Eur Phys J Appl Phys.

[14] Werzer O, Stadlober B, Haase A, Oehzelt M, Resel R (2008). Full X-ray pattern analysis of vacuum deposited pentacene thin films. Eur Phys J B.

[15] Tawa K, Kuboyama N, Ahmed SA, Tanaka M, Nakaoki T (2009). Sensitive detection of a pseudo-polyrotaxane ultrathin film by SPR and QCM-D methods. Sensors Actuators B Chem.

[16] Mohan T, Spirk S, Kargl R, Doliska A, Ehmann HMA, Kostler S (2012). Watching cellulose grow - kinetic investigations on cellulose thin film formation at the gas-solid interface using a quartz crystal microbalance with dissipation (QCM-D). Colloids Surf A.

[17] Doliska A, Strnad S, Stana J, Martinelli E, Ribitsch V, Stana-Kleinschek K (2012). In vitro haemocompatibility evaluation of PET surfaces using the quartz crystal microbalance technique. J Biomater Sci Polym Ed.

[18] Rodahl M, Höök F, Fredriksson C, Keller CA, Krozer A, Brzezinski P (1997). Simultaneous frequency and dissipation factor QCM measurements of biomolecular adsorption and cell adhesion. Faraday Discuss.

[19] Sauerbrey G (1959). Verwendung von Schwingquarzen zur Wägung dünner Schichten und zur Mikrowägung. Zeitscrift für Physik.

[20] Parratt LG (1954). Surface studies of solids by total reflection of X-rays. Phys Rev.

[21] Nevot L, Croce P (1980). Characterization of surfaces by grazing X-Ray reflection - application to study of polishing of some silicate-glasses. Rev Phys Appl.

[22] Taniguchi M, Belfort G (2002). Correcting for surface roughness: advancing and receding contact angles. Langmuir.

[23] Lafaurie A, Azema N, Ferry L, Lopez-Cuesta JM (2009). Stability parameters for mineral suspensions: improving the dispersion of fillers in thermoplastics. Powder Technol.

[24] Kiessig H (1931). Interference of X-rays in thick layers. Ann Phys Berl.

[25] Ehmann HM, Zimmer A, Roblegg E, Werzer O (2014). Morphologies in solvent annealed clotrimazole thin films explained by Hansen-solubility parameters. Cryst Growth Des.

[26] Gordon RE, VanKoevering CL, Reits DJ (1984). Utilization of differential scanning calorimetry in the compatibility screening of ibuprofen with the stearate lubricants and construction of phase diagrams. Int J Pharm.

[27] Liua D, Feib X, Wanga S, Jianga T, Sua D (2006). Increasing solubility and dissolution rate of drugs via eutectic mixtures: itraconazole–poloxamer188 system. Asian J Pharm Sci.

[28] Wypych G (2004). Handbook of plasticizers.

[29] Yang ZH, Clough A, Lam CH, Tsui OKC (2011). Glass transition dynamics and surface mobility of entangled polystyrene films at equilibrium. Macromolecules.

[30] Yang ZH, Fujii Y, Lee FK, Lam CH, Tsui OKC (2010). Glass transition dynamics and surface layer mobility in unentangled polystyrene films. Science.

[31] Tsui OKC, Wang YJ, Lee FK, Lam CH, Yang Z (2008). Equilibrium pathway of spin-coated polymer films. Macromolecules.

[32] Forrest JA, Dalnoki-Veress K (2001). The glass transition in thin polymer films. Adv Colloid Interface.

[33] Van Eerdenbrugh B, Baird JA, Taylor LS (2010). Crystallization tendency of active pharmaceutical ingredients following rapid solvent evaporation–classification and comparison with crystallization tendency from undercooled melts. J Pharm Sci.

